# Genetics and Personal Insurance: the Perspectives of Canadian Cancer Genetic Counselors

**DOI:** 10.1007/s10897-015-9841-9

**Published:** 2015-05-01

**Authors:** Michelle Lane, Ida Ngueng Feze, Yann Joly

**Affiliations:** Maritime Medical Genetics Service, IWK Health Center, Halifax, NS Canada; Centre of Genomics and Policy, Department of Human Genetics, Faculty of Medicine, McGill University, Montreal, QC Canada

**Keywords:** Canada, Cancer genetics, Genetic counseling, Genetic discrimination, Personal insurance

## Abstract

Genetic discrimination in the context of genetic testing has been identified as a concern for symptomatic and asymptomatic individuals for more than three decades. Genetic counselors are often the health care professionals who discuss risks and benefits of genetic testing with patients, thereby making them most appropriate to address patient concerns about genetics and personal insurance (i.e., life, life as related to mortgage or group insurance, disability, critical illness and travel). A pilot study was conducted to ascertain the current practices of Canadian cancer genetic counselors in regard to their discussions with patients about genetic testing and access to personal insurance. Among the 36 counselors surveyed, 100 % reported discussing the issue of genetic testing and personal insurance with their patients. Several factors influenced the content, depth and length of these discussions including age, cancer status, family members, and patients’ current and future insurance needs. Counselors reported discussing with patients the possible impact of genetic test results on access to personal insurance, possible access and use of patient genetic information by insurance companies, and whom patients should contact if they have additional questions. The most commonly reported inquiries from patients included questions about the possible impact of genetic testing on their ability to obtain insurance, and the insurability of family members. While 28 % of counselors reported having been contacted by an insurer requesting access to patient information, only one counselor was aware of or could recall the outcome of such a request. This pilot study revealed that issues concerning genetics and personal insurance are commonly discussed in Canadian cancer genetic counseling sessions. Counselors furthermore expressed a need for additional educational resources on the topic of genetics and personal insurance for themselves and their patients.

## Introduction

The use of genetic information by third parties for non-therapeutic purposes is one of the major risks associated with genetic testing and genetic research (Huizenga et al. 2010; Joly et al. 2010; Taylor et al. 2008). The phenomenon of genetic discrimination (hereafter “GD”) was introduced in scientific literature in the 1980s. One of the first empirical studies on the topic by Billings et al. (1992) suggested that symptomatic and asymptomatic individuals receiving genetic counseling services could be at risk for GD. The study defined GD as “discrimination against an individual or against members of that individual’s family solely because of real or perceived differences from the ‘normal’ genome of that individual” (Billings et al. 1992, p. 477). Since then, several definitions of GD integrating concepts from various fields of academic expertise (e.g., scientific, legal, ethical, and actuarial) have been proposed (Joly et al. 2013).

Interestingly, the significant concerns raised by GD might not be proportionate to actual risk. Documented incidents of coverage denial, or increased premiums on the basis of genetic information remain limited to a few relatively well known, highly penetrant, familial, adult-onset, genetic conditions (Joly et al. 2013). Nonetheless, some patients decline to undergo clinical genetic testing (Armstrong et al. 2003; Godard et al. 2007), seek genetic counseling services (Bower et al. 2002; Geer et al. 2001), or participate in genetic research (Kirkland et al. 2009) because of insurability concerns related to GD. To address these concerns, many countries have adopted laws or moratoria to prevent insurers from accessing genetic information (Rothstein and Joly 2009).

In the United States (US), the Genetic Information Nondiscrimination Act (GINA) was adopted in 2008 to prohibit GD in health insurance and employment (Rothstein 2009). Aware of the limited scope of this Act, the U.S. National Society of Genetic Counselors (NSGC) issued Cancer Practice Guidelines, which recommend that the possibility of GD be discussed during cancer genetic testing sessions to promote informed consent (Hadley et al. 2003). The NSGC has also recommended that patients be informed of the limitations of GINA in addressing potential GD impacts on access to life, long-term, and disability insurance.

In Canada, where no specific law addressing GD has been enacted, studies have focused on patients’ perspectives on GD in the context of highly penetrant, familial, adult onset, conditions with a significant medical impact such as Huntington disease (Bombard et al. 2007, 2009, 2012) and avenues for policy reforms (Phoenix Strategic Perspective Inc. 2013; Riley et al. 2012). More recently, a privacy survey prepared for the Office of the Privacy Commissioner of Canada reported that if recommended to undergo genetic testing, 52 % of Canadians would be very concerned they might be asked to provide test results for non-health related purposes, such as insurance or employment (Phoenix Strategic Perspective Inc. 2013). Although the Canadian and US healthcare systems differ, GINA revitalized the Canadian debate on access to genetic information and may influence policy makers at the federal and provincial level to propose similar anti-GD bills (e.g., Proposition of a Genetic Non-Discrimination Acts) (Bill 127 2013; Bill S-201 2013). It is in this politically charged context that the Canadian Association of Genetic Counsellors (CAGC) issued a position statement on GD. In this statement, the CAGC “supports and encourages the immediate development and implementation of legislation to protect the Canadian public from unfair use of genetic test results or family history” (Canadian Association of Genetic Counsellors 2012).

Patients consider genetic counselors to be experts and value their information and support, including how to improve communication about genetics within their family, help to anticipate certain feelings and experiences from future events, and clarify values underlying decisions and attitudes (Bernhardt et al. 2000). In this role, genetic counselors will have to address the issue of GD in pre-test counseling sessions. Australian and US data support this claim, demonstrating that genetic counselors discuss the possibility of GD with patients (Barlow-Stewart et al. 2009; Hall and Rich 2000; Huizenga et al. 2010; Wertz 1998–1999). Until now, the content of these discussions in the Canadian context was largely unknown. Our survey was meant to fill this important knowledge gap, and had the following objectives: 1) explore the practices of Canadian cancer genetic counselors regarding genetics and patients’ access to personal insurance, 2) determine the factors that influence discussions on this topic, 3) document GD concerns among counselors and patients and 4) identify needs that should be met to facilitate future discussions with patients.

## Methods

### Sample and Procedures

After receiving research ethics approval from the McGill Faculty of Medicine Research Ethics Board, the research team recruited participants by contacting the CAGC and requesting that our survey be sent to their membership via its electronic mailing list (~*N* = 317). Participants were required to complete the survey within 5 weeks of receiving the initial invitation e-mail and informed consent documents. They were reminded through a second e-mail notification via the CAGC listserv 2 weeks following the initial invitation. To be considered eligible for our study, participants were required to be full members of the CAGC, currently practicing in Canada and working at least part-time in the field of cancer genetic counseling. Full members not currently practicing in Canada, as well as student members, were excluded. Participants were required to have completed a master’s program in genetic counseling, or to have achieved bachelors, masters or Ph.D. degrees in a related field. Thirty-eight responses were received; however, one participant who reported currently working in the United Kingdom was excluded from the study. One respondent consented, but did not answer any questions; therefore, the total number of included respondents was 36.

### Instrument Design

The survey (Appendix) was a semi-structured questionnaire containing both multiple choice and open-ended questions. The survey included five demographic questions and 10 open-ended questions designed to assess current practices of genetic counselors in regard to discussing the issue of genetic tests and patients’ access to personal insurance. The questions were formulated following a review of the genetic counseling literature on genetics and access to insurance. The Pfeffer et al. study, in which the researchers conducted phone interviews with cancer genetic counselors in the U.S. (Pfeffer et al. 2003), was particularly helpful in designing the instrument.

### Data Analysis

The principal investigator (YJ) and study coordinator (ML) collected the data, and all three authors (YJ, ML & INF) participated in the analysis. Inductive thematic analysis was used to analyze the data collected (Braun and Clarke 2006). Themes were independently identified across the data set and agreed upon by the authors, and discrepancies were resolved through majority vote. Codes were developed and were used to organize findings into six broad themes: (1) factors considered by genetic counselors, (2) information provided by genetic counselors to patients with respect to genetics and access to personal insurance, (3) level of comfort among counselors discussing genetics and access to personal insurance, (4) patient concerns as reported by genetic counselors, (5) genetic counselors’ sources of information, and (6) risk of insurers requesting patient information. Quantitative data were tallied and graphed, however formal statistical analyses were not conducted considering the small sample size.

## Results

### Genetic Counselors’ Demographic Information

Forty percent (12/30) of study participants reported practicing exclusively in cancer genetics. Therefore, the majority of counselors surveyed practiced in at least one other area, most of which were in the adult settings (Table 1). The most common work setting (86.2 %;25/29) was a university or academic setting. Forty percent (12/30) of respondents indicated having worked in the field for 1 to 4 years. The years of work experience ranged from less than 1 year to greater than 15 years. Though all four Canadian regions (Atlantic, Central, Prairies and West coast) were represented, Central Canada had the highest geographical representation of counselors in our study. Given that some provinces, such as Nova Scotia, have only one genetics center, broad regional categories were chosen in order to ensure anonymity of the participants. Although 80 % (24/30) of respondents indicated seeing an average of four or more patients per week, they were not specifically asked about the number of patients seen in the context of cancer genetics. For this reason, the exact number of patients seen in this setting may be lower than reported, as only 40 % (12/30) of respondents practice exclusively in cancer genetics.

**Table 1:** Respondent demographics

Characteristics	Number of participants	Percentage %
Area of practice (*N* = 30)^a^		
Cancer only	12	40.0
Research	8	26.6
Prenatal	10	33.3
Cardiac	9	30.0
Pediatrics	9	30.0
Adult	14	46.7
Psychiatric	1	3.3
Other	4	13.3
Work setting (*N* = 29)^a^		
University or academic setting	25	86.2
Physician’s private practice	1	3.4
Public medical facility	5	17.2
Other	1	3.4
Region of Canada (*N* = 30)		
Atlantic	3	10.0
Central	16	53.3
West coast	3	10.0
Prairies	8	23.7
Years of practice (*N* = 30)		
Less than 1	3	10.0
1–4	12	40.0
5–9	4	13.3
10–14	7	23.3
15 or more	4	13.3
Average number of patients/week (*N* = 30)		
0–1	2	6.7
2–3	4	13.3
4–5	7	23.3
6–7	12	40.0
8+	5	16.7

Participants were not required to answer all items, resulting in fluctuation in sample size for each question

^a^Participants were asked to indicate all areas of practices and two participants indicated two responses for work setting; therefore, total percentage appears greater than 100 %

### Discussion of Genetics and Personal Insurance

All genetic counselors reported discussing the issue of genetics and access to personal insurance with their patients (see Table 2). In addition to reporting that they discuss insurance with their patients, 42 % (15/36) of these respondents supplemented their answer with additional details. In this group, counselors elaborated to indicate that they discuss the issue with all their patients (47 %; 7/15), address this issue with all patients considering testing (20 %; 3/15), or discuss personal insurance issues with all unaffected patients undergoing predictive or asymptomatic testing (33 %; 5/15).

**Table 2:** Genetic counselors’ responses to quantitative items

Responses	Number of responses	Percentage (%)
Do you discuss genetics and access to personal insurance with patients? (*N* = 36)		
Yes	36	100
No	0	0
How often do patients initiate the conversation? (*N* = 36)		
Always	1	2.8
Most of the time	1	2.8
Some of the time	25	69.4
Seldom	8	22.2
Never	1	2.8
In general, how comfortable are you discussing genetics and access to personal insurance with patients? (*N* = 31)		
Very comfortable	1	3.2
Comfortable	16	51.6
Neutral	9	29.0
Uncomfortable	5	16.1
Very uncomfortable	0	0
Is the issue of insurer’s potential access to medical records mentioned on your center’s consent form? (*N* = 31)		
Yes	7	22.6
No	20	64.5
Uncertain	4	12.9
How would you qualify the risk of insurer requesting patients’ records, or of patients having difficulty obtaining insurance, following genetic testing? (*N* = 29)		
Very high	2	6.9
Somewhat high	8	27.6
Low	18	62.1
Almost non-existent	1	3.4
Have you ever been contacted by an insurer to provide genetic information or results about one of your patients? (*N* = 29)		
Yes	8	27.6
No	21	72.4
Uncertain	0	0

Counselors were not required to answer all items, resulting in fluctuation in sample size for each item

#### Factors Considered by Genetic Counselors When Discussing Insurance

Genetic counselors all reported discussing the issue of insurance with their patients. In addition, some counselors also described a set of factors that influenced the length and depth of their discussion and included: the patient’s age, whether the patient already had a personal history of cancer, whether or not the patient was eligible for testing, whether or not the patient had children or siblings, and whether the patient was likely to need insurance in the future. Other elements that impacted the discussion were the patient’s desire for more information and whether or not they were concerned about the effects of test results on their insurability. As explained by a genetic counselor:

What the exact conversation looks like really depends on the context in which I’m seeing a patient (i.e., are they affected with cancer, are they young and being seen for predictive testing, etc.).

Throughout the questionnaire, it was more common for counselors to mention discussing GD with patients who were currently unaffected. For example:

I tend to do it more in the context of pre-symptomatic testing. When a patient has already had cancer that in itself has bigger impact on insurability than genetic testing.

#### Information Genetic Counselors Provide to Their Patients

##### Types of Personal Insurance Most Referenced in Discussions:

When addressing the potential impact of test results on patients’ insurability, 41 specific references to certain types of insurance were made. These included life insurance (19), disability insurance (8), mortgage related insurance (5), travel insurance (5), critical illness (3), and group insurance (1). Two counselors reported mentioning health insurance and reassured patients this coverage would not be affected.

Counselors also provided examples of insurance challenges that patients could expect. One counselor stated:

I typically discuss that genetic test results may impact their ability to obtain certain types of insurance in the future. I explain that this may include being denied a new insurance policy or charged higher premiums for a new policy if they are found to carry a pathogenic mutation. Specific types of insurance I provide as examples include life insurance or disability insurance on a mortgage. I explore with them how this may or may not impact their decision about genetic testing.

##### Patients’ Disclosure of Information to Insurers:

Genetic counselors reported advising patients about their duty to disclose information to insurers.

While some counselors informed patients that they were required to answer insurers’ questions honestly, most counselors also reported advising patients that they were not required to disclose additional information beyond what insurers ask on their forms. Examples of how genetic counselors perceive the patients’ disclosure requirements include:

Patients should carefully read policies for what ongoing reporting is required but generally, one is only required to answer honestly the questions that are asked.

I also emphasize the fact that no one is obliged to provide genetic testing information unless they are asked to, and as far as I know, the question “is there a genetic condition in your family, or do you have an inherited genetic condition” is not a standard one for most companies (yet).

Insurance companies WILL use genetic test to assess their eligibility and cost. Families must answer all questions truthfully on their insurance applications, but are not required to volunteer additional information that is not requested.

Obligation to report it if the question is asked by an insurer.

One counselor noted informing patients that failing to answer questions on an insurance application form truthfully could result in the insurance contract being annulled.

##### The Impact of Family History vs. Genetic Testing:

While most genetic counselors advise their patients to secure insurance before undergoing testing, they also discuss the impact that a family history of cancer may have on their insurability and that of their family members. Numerous genetic counselors reported that their discussion with patients who have a familial history of cancer focuses on explaining that family history may have a greater effect on insurance eligibility and premiums than the results of a genetic test.

I stress that family history may determine insurance rates, regardless of whether they choose to have genetic testing or not.

Positive results may affect insurability, although family history may also have an effect regardless of GT [(genetic test)] results.

Also discuss that family history may often be as damaging as genetic testing unless the history is quite removed from them.

Their family history will have most significance to the insurance company.

I feel fairly confident that personal and family history, if striking, will trump any genetic information.

##### Information on Insurance Practice

Additional comments were made by some genetic counselors about the practices of insurance companies in Canada:

I explain that although there have been initiatives to prevent genetic discrimination in the U.S. and now in Canada, there are still means for an insurance company to decline a patient life insurance based on their genetic risk. We have no guarantee that it will be a problem for every individual.

We discuss that genetic tests are not something that all companies integrate into their evaluation, nor in the same way.

At some point our recommendations for appropriate screening may conflict with insurance interests. For example, even without genetic testing an insurance company would likely “catch on” to increase familial risk if they see a 30 year old who is getting annual breast MRI.

Insurance companies may investigate every part of a patient’s health record. Insurance companies may not insure unless the patient gets predisposition testing and is negative.

Counselors also expressed being unsure how insurers may incorporate genetic information into insurance decisions:

Uncertainty in insurance coverage—uncertainty whether cancer syndromes are diseases (question asked about disease in family)—Uncertainty whether a known mutation in a parent/sibling is considered by insurance even if [it is] not supposed to be used.—Uncertainty whether a known mutation in oneself will impact insurance decision.

Lack of definitive and shared standards between insurance companies.

It is hard to predict how an insurance company would view a personal cancer diagnosis vs. a diagnosis of a hereditary cancer syndrome.

We don’t really know how insurance companies value genetic test results.

Because all my information is anecdotal and because each insurance company will ask different questions, I really don’t know how this information may affect my patients and their families now, or down the road.

##### Referral to Other Experts

Genetic counselors reported advising patients to secure insurance before undertaking a genetic test and to speak with other professionals when patients had more questions. Fourteen counselors specified experts to whom they refer their patients. Among these, 8 specifically advised their patients to seek more information from insurance companies (including agents or brokers), 2 counselors mentioned referring patients to lawyers, and 1 advised that patients should discuss this with their physician. Counselors express referring patients in different ways:

To see if it [genetic test] would have an effect on existing policies, [I advise them to] consult their broker.

If a patient is concerned at all, I advise that he/she consult physician/lawyer/insurance agent to discuss further before initiating genetic testing.

I tell them there is a chance that they could be refused insurance based on test results, but I’m no expert in the matter, and if it’s something they feel very strong about they should consult an attorney.

Only that it could be an issue and if [there are] concerns to discuss with a broker.

I sometimes find I cannot answer their more specific questions. When that’s the case, I typically encourage patients to gather more information by contacting insurance companies prior to having testing done.

#### Levels of Comfort Discussing GD

**Fig. 1:**
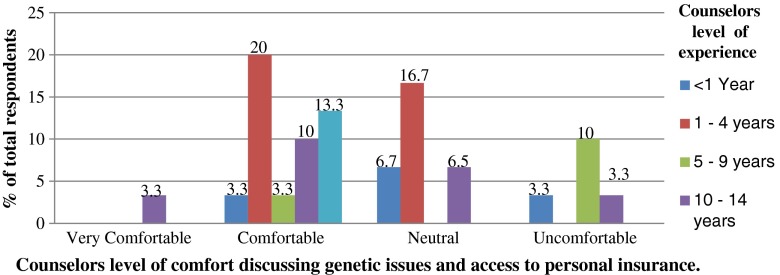
Reported level of comfort of counselors in discussing genetics and patients’ access to personal insurance. *(N = 30)* ^a^. ^a^One counselor who reported feeling comfortable did not provide information regarding years of experience

The level of comfort of counselors in discussing issues of genetics and access to personal insurance with patients was also explored. Overall, among the 31 counselors who shared their level of comfort, 16 counselors reported feeling comfortable, 9 neutral and 5 uncomfortable. While no one reported feeling very uncomfortable, 1 said feeling very comfortable (Table 2; Fig. 1).

Although a large proportion of counselors 25/31 (79.8 %) reported feeling comfortable or neutral discussing this issue with patients, some stated they are often unable to answer patients’ questions. For example, one counselor stated:

I feel somewhat comfortable discussing these issues with patients, although, I sometimes find I cannot answer their more specific questions. (Reported comfort level: Neutral)

One genetic counselor reported feeling comfortable discussing the issue with patients in spite of being unable to answer specific questions:

Again, I can only tell patients general information that I am familiar with. Details are specific to each person and their own situation. For exploration of that, people need to speak with a professional [with expertise in insurance]. (Reported comfort level: Comfortable)

In contrast, 5/31 (16.6 %) of counselors revealed feeling uncomfortable discussing the issue of access to personal insurance with patients. One, who reported feeling uncomfortable, described lacking information in regard to the topic, as well as experiencing a challenge in presenting information in a balanced manner:

I do not feel like I have any real answers to patient questions. Each individual’s plan may be different. This is a common concern for patients referred to our clinic, but I do not get much feedback from families whether or not they actually did have trouble obtaining insurance. I worry about balancing a potential financial risk vs the medical benefit in knowing about cancer risks. (Reported comfort level: Uncomfortable)

Some counselors pointed out they have a duty to address the issue of genetics and personal insurance with patients regardless of their professional ability to provide concrete evidence:

I don’t know what concrete information to give patients or even if I should be giving information to patients at all. Even though I don’t know the facts, insurance is something I feel I have a duty to bring up along with other topics that patients may not otherwise think about when considering genetic testing. (Reported comfort level: Uncomfortable)

In addition, counselors made a point to let patients know that they lack the expertise needed to fully address issues related to genetics and insurability as seen in the following two examples:

I reiterate that I am NOT an insurance expert.

If a patient asks me pointed questions I am quick to say that I am not the best person to talk to as I have no training on this subject.

#### Patient Concerns as Reported by Genetic Counselors

Counselors indicated that patients sometimes initiate the conversation about genetics and access to personal insurance. Thirteen counselors also reported that patients raised concerns about certain types of personal insurance including life insurance (5/13), travel insurance (4/13), disability (2/13), critical illness (1/13), and mortgage (1/13).

Counselors were asked to comment on the most common questions and/or concerns expressed by patients with respect to insurability. Almost 69 % (20/29) of counselors conveyed that patients most commonly inquire about whether genetic test results will impact their insurance eligibility and insurance premiums. Additionally, 3 counselors described questions patients asked them concerning whether genetic test results would impact their current insurance policies. This following comment illustrates this concern:

Does having genetic testing mean I will not be able to get insurance or will my current policy be cancelled?

Furthermore, surveyed counselors often communicated that patients were concerned about the potential impact of their genetic test results on family members who may seek personal insurance, including children and siblings:

Will they be able to get insurance (this has included life, disability, critical illness, and travel health insurance)? Will it affect their children’s ability to get insurance? At what point is there a risk to their children’s ability to obtain insurance?

The results suggest that patients seek help from genetic counselors to better understand the current practices of the Canadian insurance industry. Three counselors noted patient questions regarding the confidentiality, storage and disclosure of genetic test results. As outlined in the following statements, one respondent believed patients who decline a referral for genetic counseling might do so out of fear for their insurability:

I do not have numbers, but concern about insurance for self or family seems to be a common reason that people give if they decline a referral to our cancer genetics program.

This is a common concern for patients referred to our clinic, but I do not get much feedback from families whether or not they actually did have trouble obtaining insurance.

Counselors expressed difficulty in providing answers to patients’ specific questions regarding insurance practices in Canada. More specifically, they were unable to predict the likelihood of insurers requesting patient genetic information, the rate of increased patient premiums, or the degree of insurance coverage denials. In such cases, one counselor reported:

I always fall back to the safest choice, which is, if considering purchasing insurance, do it before you know what your genetic status is.

#### Genetic Counselor’s Sources of Information

Counselors reported relying on information gathered from discussions with their colleagues, as well as past patient experiences. Other sources of information they sought included medical literature and professional reports (from institutions such as the Canadian Actuaries Institute), personal experiences, presentations from professional conferences, teaching during their training programs, and websites (Table 3). Three counselors conveyed they did not use any specific sources of information.

**Table 3:** Sources of information reported by counselors. *(N = 28)*

Source	Number of responses	Counselors comments
Discussions with other genetic counseling colleagues	11	*Rely heavily on discussions that take place in the counseling community (ex. through CAGC listserv)…*
Patient experiences	10	*Patients’ experience with insurance*
Professional association conferences/presentations	7	*Information from presentations by insurance brokers at recent genetic counseling conferences.*
Personal experiences	5	*I have personal experience and anecdotal evidence.*
Information during training program	5	… *lectures from an underwriter during my training*
Published reports/literature	4	*Canadian genetics and life insurance task force report*
No specific resources	3	*I don’t have any sources of information that I rely on to inform counselees about genetics and access to personal insurance.*
Websites	1	*Websites (ex Canadian coalition for genetic fairness)*

One counselor who reported not using any specific sources of information expressed, with others, a desire for access to better resources as this issue can frustrate the consultation process:

I wish I had access to better sources.

This is something I have expressed with colleagues that we all need more info on. Most of us just seem to hand wave and don’t have concrete documents/resources to rely on or share with patients/families.

I feel there is extremely limited information and nobody really knows anything and so the discussion is very speculative. This makes it hard for patients to make any kind of informed decision.

This question is good- it made me realize that a short guide for GCs [Genetic counselors] would be very helpful.

One counselor described using available literature on GD from other countries as sources of information. A need for specific resources on the issue of genetics and access to personal insurance for Canadian counselors was reported by three other counselors throughout the study. One counselor expressed that a short course was needed to further educate genetic counselors on this issue.

#### Request for Patient Genetic Information from Insurers

##### Risk of Insurance Companies Requesting Genetic Information

Counselors in our study reported telling their patients how little is known about how frequently insurance companies request genetic test results, and how those results may impact patient insurability.

I have never been requested to write a letter to a company on behalf of a patient, but can imagine how a genetic counsellor’s letter could assist the company in making an accurate risk assessment (e.g., healthy patient who carries a MLH1 mutation and is compliant with annual colonoscopy).

**Fig. 2:**
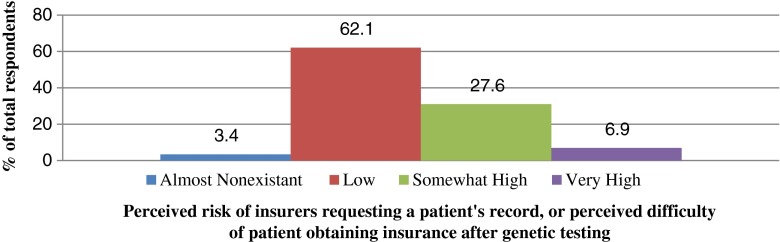
Counselors’ perceived risk of insurers requesting patient information. *(N = 29)*

When surveyed to qualify the risk of insurers requesting patient information, 18/29 (62.1 %) counselors estimated it to be *low*, 8/29 (27.6 %) find it *somewhat high*, 2/29 (6.9 %) *very high*, and 1 (3.4 %) responded *very low* (Fig. 2).

##### Patients’ Consent to Insurers and General Practitioners’ Access to Their Genetic Results:

Counselors were also asked whether an insurer had contacted them to request patient records. Eight of 29 respondents (27.6 %) reported an insurer had contacted them under different circumstances (i.e., when a patient was applying for insurance, or after a claim had been submitted). Of these 8 counselors, one did not provide any details on the specific incident, and another could not recall the full circumstances. Two counselors reported being contacted about neurogenetic patients, but not for cancer genetic patients. One counselor described being contacted very seldom, and most often for patients who tested negative for a cancer predisposition gene:

Usually the patient has signed a consent form for the insurer to get the records. It has mostly happened for individuals proving that they do NOT have the condition in the family.

Another counselor described verifying that patients had actually consented to have their results disclosed to the insurer:

In all cases, the patients have provided the insurance company with our program details, and I have always double checked this request contacting the patient to advise them that I had received such a request.

The majority of counselors (20/30; 66.7 %) stated that the possibility that insurers could have access to patient genetic data was not mentioned on the genetic test or the research consent forms at their center.

##### Instances Where Counselors Were Requested to Provide the Genetic Information of Patients:

One counselor noted being contacted by an insurer once in 10 years of practice. This particular event involved an unaffected *BRCA* gene mutation carrier, but no additional information was provided by the counselor. Another counselor was asked to provide a letter on behalf of a patient to indicate that genetic testing was not offered due to the patient’s low risk of carrying a cancer predisposition gene mutation. Another counselor shared being contacted by insurers on several occasions, most often to verify a patient’s negative genetic test result. This counselor went on to describe an event whereby both the patient, and the family member were affected by the contact with an insurance company. The details of the event are presented in the following comment:

The original patient was being promoted at work, and in the processing of her benefit package, they obtained her consult[ation] letter from genetic counseling through the GP files. The insurance provider communicated that they would defer her processing until the results of her genetic testing were confirmed. The mother [of this patient] was too afraid to ever come in for counseling and testing, and the patient dropped out of communication with our clinic.

The same counselor also described sending a letter on behalf of a patient who was a *PMS2* gene mutation carrier but was believed to have a low penetrance allele.

In these examples, information on the patient’s insurance outcome was unknown. Nevertheless, one counselor shared the outcome of such an inquiry:

I was contacted by an insurance company because a patient of mine had disclosed that she’d had an appointment with genetics (I don’t know if she volunteered this information, or was asked outright).(…) I got in touch with her [the patient] about this issue, and she asked me to send a consult letter to the insurance company, to reassure them that we did not feel her risk to develop breast cancer was greatly increased over the population, which I did. It seemed that information from our clinic helped with her insurance application.

## Discussion

This study explored the practices of Canadian cancer genetic counselors regarding genetics and personal insurance. Our results provide insight into Canadian cancer genetic counselors’ perceptions regarding genetic testing and access to personal insurance in the Canadian genetic counseling profession. Our results could also be used to better inform current federal and provincial debates surrounding the need for a legal protection against GD in Canada (Bill 127 2013; Bill S-201 2013).

Counselors were surveyed about (1) the factors they take into consideration when discussing GD and insurance, (2) the content of the information they provide to patients, (3) their perspectives and level of comfort discussing GD, (4) patient concerns, (5) their sources of information, and (6) requests from insurers to access patient genetic information.

All 36 genetic counselors declared discussing issues related to genetic information and access to personal insurance with their patients, underlining that this particular topic is an important concern in their practice. This is consistent with findings from a similar empirical study by Wham et al. (2010), where 75 % of the 150 counselors surveyed declared discussing risks of insurance or employer discrimination during their initial cancer genetics consultation visits. The fact that patients sometimes initiated such discussions may reflect patients’ concerns about issues on genetics and access to personal insurance. This is consistent with the findings of other studies describing the potential for GD in the context of insurance before the adoption of specific legal protections against GD in the US (Bower et al. 2002; Hall and Rich 2000).

### Factors Considered by Genetic Counselors When Discussing Insurance

All counselors reported a number of patient- and context-specific characteristics influenced the depth and lengths of their discussions with patients. These factors included the patient’s health and family status, need for insurance and individual concerns. Our findings contrast with those from Pfeffer et al. (2003), where only 16 % of surveyed NSGC counselors mentioned taking into account patients’ characteristics as factors influencing the extent of their discussion on genetic discrimination. We note however, that the Pfeffer et al. study was based on a large US sample, and thus likely to reflect different insurance concerns such as health insurance, particular to the US context.

### Information Provided by Genetic Counselors

Canadian counselors provide a range of information from which five main subjects were identified: (1) the types of insurance most referenced in discussions, (2) patient’s requirement to disclose information to insurers, (3) the potential impact of family history in contrast with genetic test results, (4) perspectives and information on insurance practice, and (5) referral to other professionals.

#### Types of insurance referenced most in discussions with patients

In the US, where health insurance is not universal, fear and concerns of GD in the context of health insurance was an important concern reflected through the adoption of GINA, a legislation restricted to addressing GD in the contexts of health insurance and employment (Hall and Rich 2000; Ragoussis et al. 2014). However, in the Canadian context—where health insurance is universal—it was not surprising to find that genetic counselors discussed life insurance most with patients. This finding is in line with current international concerns about genetic discrimination in the context of life insurance (Joly et al. 2013; Otlowski et al. 2012). Nonetheless, we note that counselors reported travel insurance was the second most cited type of insurance patients identified as source of concerns. This is a very interesting finding given the current dearth of academic research on the use of genetic information for this particular type of insurance contract.

#### Patient’s duty to disclose information to insurers

Counselors provided varying information on the duty of patients to disclose information to insurers. While most counselors informed patients that they were required to answer the insurers’ questions truthfully, some counselors defined this duty to be limited to the insurance questionnaire by informing their patients that they were not required to “volunteer” genetic information unless asked by the insurer. This interpretation of the legal requirements of disclosure could mislead or be misinterpreted by patients to mean that they are not required to disclose all information that is material to their insurance coverage eligibility (or insurability). Indeed, in Canada, a contract for personal insurance is based on the principle of “good faith” (utmost good faith in Quebec civil law) whereby an applicant is required to disclose “all the facts known to him which are likely to materially influence an insurer in the setting of the premium, the appraisal of the risk or the decision to cover it” (Quebec Civil Code 1991, Art. 2408), while in turn, the insurer is required to provide a coverage amount based on the fair assessment of the applicant’s risk (Canadian Life and Health Insurance Association Inc. 2010; Gregoire et al. 2009). Applicants who fail to comply with this legal obligation risk seeing their life insurance contract annulled by the courts at the request of the insurer (*Audet v. Industrielle-Alliance* (Quebec) 1990; Pfeffer et al. 2003). Therefore, while patients may benefit from some information about insurance policies applicable to genetic information (Trepanier et al. 2004), counselors must be very cautious when discussing patients’ legal rights and obligations. Providing patients imprecise, inaccurate or incomplete information could have legal repercussion on patient’s insurance contracts, and could even result in their policy being cancelled. We also note that the challenges with counselors being aware and understanding the full requirements and limitation of the laws within their jurisdiction is not unique to Canada but has also been documented in the US. For example, despite the adoption of GINA in the US, many genetic counselors are still not fully aware of the law and its limitations (Pamarti 2011).

#### Impact of family history of diseases in contrast with genetic test results

Generally, counselors reported advising patients that family history of diseases may have a greater impact on their insurability than genetic test results. Indeed, detailed family histories of diseases have been considered an important source of genetic information by insurers which, in some cases, may constitute a more accurate prediction of future health than the results of many current genetic tests (Hudson et al. 2007). It is interesting to note that some of the countries that have adopted laws to restrict access to genetic test results still authorize insurers to use information about family history for underwriting (Lemmens 2004). The current *Bill on Genetic Discrimination* (S-201) introduced in the Canadian Senate is one example. It focuses on restricting access to information from “genetic testing,” but seems to exclude family history from its scope of application (Bill S-201). Genetic counselors should keep informed of legal developments on genetics and insurance in Canada to better inform their patients (Trepanier et al. 2004). This is especially important given the position of the CAGC in favor of a legal protection against GD (Canadian Association of Genetic Counsellors 2012).

#### Information on insurance practice

In our study, counselors commonly recommended that patients secure all insurance needs before undergoing genetic testing. Indeed, the Canadian life and health insurance association (CLHIA) stated that while they would not require applicants to undergo genetic testing, they would seek access to genetic test results whenever they have been made available to the applicant or her/his physician (Canadian Life and Health Insurance Association Inc. 2010). Nevertheless, as one counselor mentioned, insurers may still be able to determine patients’ genetic profile based on other elements in their medical records or family history. Genetic counselors were generally uncertain about how insurers used genetic information when assessing patients’ insurance coverage eligibility.

#### Referral to other professionals

Our findings indicate that counselors informed their patients that they do not have expertise on issues concerning genetic testing and personal insurance. Rather they recommended patients seek the assistance of other professionals including insurance agents, physicians, and lawyers. Given their vested interest in using genetic information, insurance representatives may not be a completely impartial source of information for patients. Conversations with physicians and lawyers may be more advantageous considering available confidentiality and professional secrecy protections. Nevertheless, physicians, like genetic counselors, may also lack the appropriate expertise on personal insurance, and an attorney can be costly even for a single consultation. This underlines an important need for more training in this area, and a need for additional resources for patients.

### Genetics Counselors’ Source of Information

Our findings demonstrate that counselors would welcome additional resources on genetics and personal insurance to facilitate their discussions with patients. As reported, most counselors rely on their discussions with patients or colleagues as sources of information regarding genetics and personal insurance. Alternatively, some do not use any reliable sources or use literature from foreign countries. While the use of international literature may provide counselors with a global perspective on issues relevant to their practice, great caution should be exercised as such information may not be applicable to Canadian healthcare contexts. Indeed, countries have different conceptions of social, economic and human rights entitlement and have adopted a variety of approaches to address concerns about genetics and personal insurance (Joly et al. 2010).

This pilot study highlighted an important knowledge gap among genetic counselors, and identified a need to provide genetic counselors with resources that include more complete, accurate and accessible information on genetics and personal insurance. This could take the form of a pamphlet or a training course, which should include up-to-date information on insurance underwriting practices, familial implications, summary data on the use of genetic data for insurance in Canada, applicable laws and recent policy developments. Based on the findings of this study and the lack of available resources on this subject, we recommend that additional resources be developed for patients, which should seek to address the following four main concerns: (1) the potential impact of genetic test results on insurability, (2) the patient’s duty to disclose information material to the insurer, (3) the state of the current evidence on genetic discrimination, and (4) additional information as to whom to contact for supplementary information or to discuss specific concerns.

### Level of Comfort of Counselors Discussing Genetics and Access to Personal Insurance

While genetic counselors acknowledged their limited awareness of the policies and laws applicable to insurer’s practices and expressed a need for additional resources, they often reported feeling comfortable or neutral when discussing these issues with patients. Although this may at first appear contradictory, genetic counselors, like other health care professionals, are often called to discuss with their patients matters involving some degree of uncertainty (Parascola et al. 2002). These conversations may include uncertainty about whether or not a mutation will be detected, if patients will develop cancer, if they will be identified as a carrier of a particular cancer predisposition or gene mutation. However, the issue of genetic testing and insurance carries significant legal implications that may be overlooked or misinterpreted by counselors lacking appropriate knowledge or understanding of the law.

### Patient Concerns as Reported by Genetic Counselors

According to counselors in our study, reports on patients’ concerns place travel insurance as the second most cited type of personal insurance after life insurance. Issues concerning travel insurance have been overlooked and may warrant further exploration. This issue is especially pressing given that travel insurance may be required not only for vacation travel, but also to study or work abroad, both of which are considered a growing competitive asset when seeking employment (Calleja 2012; Stone and Petrick 2013).

### Risk of Insurers Requesting Patients’ Information

The majority (18/29; 62.1 %) of Canadian cancer genetic counselors surveyed estimated that the risk of insurers requesting patient information or the risk that a patient would experience difficulties obtaining insurance following genetic testing was low. Nonetheless, 10/29 (34.5 %) perceive such risk to be somewhat high or very high. These findings are lower than results from studies conducted in the US. For example, in 2003 (before the adoption of GINA) as many as 82 % of NSGC had estimated that such risk was low (Pfeffer et al. 2003), whereas a post-GINA survey reported that 94 % of NSGC consider the risk of genetic discrimination to be low or theoretical (Huizenga et al. 2010). Thus, it appears that Canadian cancer genetic counselors have greater concerns about their patients’ insurability risk than their American colleagues did before and after the adoption of GINA.

The risk that patients may be treated unfairly due to their genetic test results is an ethical and professional challenge for genetic counselors (Bower et al. 2002). Counselors’ perception of GD risks may influence how they view coping strategies against it (Bombard et al. 2013), the content of these discussions with their patients (Pfeffer et al. 2003) or their level of comfort discussing these issues. It is worth noting that, among the 8 counselors who estimated the probability of an insurer to request patients’ genetic information as somewhat high, 6 had never been contacted by an insurer. The only counselor who estimated this risk to be high reported never having been contacted by insurers. These findings are in line with the conclusions drawn in a recent systematic review revealing that the actual risk of GD in the context of life insurance is low, while public concerns of this risk are substantial (Joly et al. 2013).

While 8/19 (28 %) of counselors reported having been contacted by an insurer requesting access to a patient’s genetic information, in only one instance were specific details about the final outcome of such inquiry provided. Only one counselor shared details of the contact, and that exchange with the insurance company ultimately enabled the patient to secure insurance. Greater knowledge of patients’ insurance outcomes following insurers’ access to genetic data is needed in order to objectively evaluate the real impact of genetic discrimination. Further studies in this regard are warranted. Considering current waitlists for cancer genetic counseling across Canada, counselors’ workload is unlikely to allow them to document such requests and to follow-up with patients concerning final outcome with insurance. Yet, the CAGC could play an instrumental part in this assessment by developing and maintaining a Canadian repository to which patients could directly report when they are experiencing difficulties obtaining and maintaining insurance following genetic counseling services or genetic testing.

Currently in Canada, insurers are permitted to use relevant genetic information in order to process coverage application and claims. According to the CLHIA, insurers may seek access to existing genetic test results when available to the patient or his physician (Canadian Life and Health Insurance Association Inc. 2010, 2014). However, in order to obtain personal health data, including genetic information, insurers are required to seek the applicant’s consent, which is often done through a data access and sharing clause included in the insurance application form (Ngueng Feze and Joly 2014). Despite the fact that insurers may present counselors with a patient’s written consent agreement to access their genetic data, one counselor mentioned always contacting patients to ensure they were aware the request has been received, and verified the patient’s consent before sending the information. Another counselor mentioned that patients at their center had the option of restricting access to their genetic data. Previous findings attest to the ethical importance both counselors and patients attribute to confidentiality, and associated safeguards should be included in discussions with patients (Bower et al. 2002; Trepanier et al. 2004).

Finally, the majority of counselors surveyed (20/30; 66.7 %) stated that the possibility of insurers’ accessing patient’s medical records (including genetic information) was not mentioned on the genetic test and/or on the research consent forms at their center. This may reflect an overall opinion in the medical community that the risk of insurer’s requesting such access is very low. Indeed, studies have demonstrated that despite the lack of evidence of genetic discrimination in Canada, Canadians in general, patients and research participants in particular, were likely to decline genetic testing when informed of potential impacts to insurability (Godard et al. 2007; Phoenix Strategic Perspective Inc. 2013). An Australian study came to similar conclusions, where the proportion of participants who declined genetic testing among those informed of insurance implications was more than double the proportion among those without such knowledge (Keogh et al. 2009). Access to genetic test results remains a subject of debate in Canada. For example, while the Canadian Institute of Actuaries states that “if relevant genetic test results are available to an individual applicant, the results must be shared with the insurer in order to preserve the integrity and proper functioning of the insurance mechanism”, the Office of the Privacy Commissioner of Canada, which oversees the Personal Information Protection and Electronic Documents Act (PIPEDA) estimates that “it is not clear that the collection and use of genetic test results by insurance companies is demonstrably necessary, effective, proportionate or the least intrusive means of achieving the industry’s objectives at this time” (Canadian Institute of Actuaries 2014; Office of the Privacy Commissioner of Canada 2014).

## Considerations for Practice and Future Directions

Through this pilot study, genetic counselors clearly identified a need to better understand the context and issues associated with the use of genetic data by insurers to increase their knowledge and better assist patients. Although genetic counseling sessions may vary in content, depth and length, it is important that patients be provided with accurate and appropriate information related to genetics and personal insurance that is consistent across cancer genetic services. Such information is a pre-requirement to ensure that patients can make an informed decision about undergoing genetic testing for hereditary cancers. Our study identified a need for additional resources in order to improve current practices. To this end, an additional course or session on this issue could be incorporated in the genetic counselors’ academic curriculum, on a recurring basis at conferences or workshops at the CAGC’s meetings (or as part of an ongoing continued professional education). Given that certain provinces have a limited number of genetic counselors, identifying an experienced counselor or another resource person within the structure of the CAGC will provide access to valuable information and support, especially for isolated practitioners. In addition, a pamphlet or another type of information document presenting background information and discussing current Canadian challenges on genetics and personal insurance could also be of assistance to counselors, who could convey the information in layman’s term to their patients during genetic counselling sessions. The use of these additional tools should be monitored to properly ascertain their usefulness and efficiency. Given that this study focused on cancer genetic counselors and that most participants also provide counseling in other genetic fields, it is quite possible that all Canadian genetic counselors would stand to benefit from the aforementioned additional resources.

Moreover, most counselors reported not knowing the outcome of cases where insurers had requested access to the patient’s genetic information. This represents an important knowledge gap, the improvement of which could assist counselors in better understanding whether negative outcomes are frequent or not. Thus, the CAGC could, with the consent of the patients, monitor these situations and document the outcome of such cases. This information could be aggregated with the national association data or used as metrics in future GD studies.

Finally, this pilot study provided an appreciable sample of qualitative data to be used as a foundation for larger Canadian studies. An interesting element that was not assessed in our pilot study was where counselors received their training (whether in Canada or abroad), in order to evaluate whether this may have impacted their knowledge about personal insurance. There is also a need to further explore counselors’ knowledge (including information needs), practice and concerns related to the use of genetic information by other third parties. For example, further studies could expand beyond cancer genetics to include a broader range of counselors, and incorporate mixed method study instruments including both qualitative and quantitative data.

## Limitations of the Study

Although this study is the first to focus on the views and experiences of Canadian cancer genetic counselors, there are a few limitations that should be taken into account when interpreting our findings. First, this pilot represents a small sample size of counselors, among whom many did not practice exclusively in cancer genetic counseling. Therefore, the perception and practices of counselors who declined participation may have differed. The number of respondents is representative of cancer genetic counselors in Canada, which make up a small community generally. Second, the study instrument requires further validity and reliability testing. Since all data were self-reported, further qualitative or quantitative studies with larger populations of counselors and patients could help to contextualize our findings and improve survey design.

## Appendix

### Study Instrument

1. Do you discuss genetics and access to personal insurance with your patients?
  - Yes
  - No
    If Yes, please specify
2. How often do patients initiate the conversation about genetics and access to personal insurance?
  - Always
  - Most of the time
  - Some of the time
  - Very seldom
  - Never
3. What factor(s) do you consider when determining whether or not to discuss genetics and access to personal insurance?
4. What information do you provide to your patients with respect to genetics and access to personal insurance?
5. What source(s) of information do you rely on to inform counselees about genetics and access to personal insurance?
6. What are the most common questions and/or concerns expressed by patients with respect to genetics and access to personal insurance?
7. In general, how comfortable are you discussing genetics and access to personal insurance with patients?
  - Very comfortable
  - Comfortable
  - Neutral
  - Uncomfortable
  - Very uncomfortable
    Please specify:
8. Is the issue of insurers’ potential access to medical records mentioned on the genetic testing and/or research consent forms at your center?
  - Yes
  - No
  - Uncertain
9. How would you qualify the risk of insurers requesting patients’ records, or of patient’s having difficulty obtaining insurance, following genetic testing?
  - Very high
  - Somewhat high
  - Low
  - Almost nonexistent
10. Have you ever been contacted by an insurer to provide genetic information or results about one of your patient?
  - Yes
  - No
  - Uncertain
    If yes, please specify:
11. In what area(s), other than cancer genetics, do you practice? Please circle ALL applicable answers.
  - Cancer Genetic Only
  - Research
  - Prenatal
  - Cardiac
  - Pediatric
  - Adult
  - Psychiatric
  - Specialty Clinic (please specify): ________________
12. Which of the following best describes your work setting?
  - University or academic health center
  - Private medical facility
  - Public medical facility
  - Physician’s private practice
  - Laboratory
  - Other (please specify): ______________
13. In what region of Canada do you practice?
  - Atlantic
  - Central
  - Prairies
  - West Coast
14. For how many years have you been providing genetic services?
  - <1
  - 1–4
  - 5–9
  - 10–14
  - ≥15
15. On average, how many patients do you counsel in 1 week?
  - 0–1
  - 2–3
  - 4–5
  - 6–7
  - 8+
